# Dr. J. Lewis Smith

**Published:** 1897-07

**Authors:** 


Dr. J. Lewis Smith died at his residence in New York, June 9, 1897, aged 69 years. He was born at Spafford, N. Y., October 15, 1827; graduated at Yale in 1849; studied medicine with Drs. Caleb Green and Frederick Hyde; attended at Buffalo Medical College in 1851 and 1852, and graduated from the College of Physicians and Surgeons in 1853. Dr. Smith was one of the first in this country to pursue the special study of diseases of children, in which department he became an acknowledged authority. In 1869 he published a treatise on the subject, the first to appear in America, which has since passed through many editions and which has been adopted as a text-book in the majority of medical schools in the United States.

Dr. Smith has been an industrious writer, contributing constantly to the periodical medical literature—annuals, journals and systems of medicine. For many years he was professor of diseases of children at Bellevue Hospital Medical College; attending physician to Charity Hospital, the New York Foundling Asylum, and the New York Infant Asylum; and consulting physician to the department for diseases of children at the Bellevue Bureau for relief of out-door poor. He was a brother of Dr. Stephen Smith, the eminent surgeon. Though he has not been in robust health for some years he was still able to practise his profession until quite recently. Several years ago he was prostrated by a severe shock from a runaway accident and a few weeks ago he met with a cable car accident which confined him to his bed. Death finally resulted from cardiac exhaustion.

